# Non-synaptic Mechanism of Ocular Dominance Plasticity

**DOI:** 10.1101/2025.09.02.673699

**Published:** 2025-09-07

**Authors:** Maxwell K. Foote, William C. Huffman, Erin N. Santos, Philip R. Lee, Michal Jarnik, Wei Li, Juan S. Bonifacino, R. Douglas Fields

**Affiliations:** 1 Section on Nervous System Plasticity and Development, *Eunice Kennedy Shriver* National Institute of Child Health and Human Development, National Institutes of Health, Bethesda, MD 20892; 2 Retinal Neurophysiology Section, National Eye Institute, National Institutes of Health, Bethesda, MD 20892; 3 Section on Intracellular Protein Trafficking, *Eunice Kennedy Shriver* National Institute of Child Health and Human Development, National Institutes of Health, Bethesda, MD 20892; 4 Oregon Health & Science University, Portland, OR 97239; 5 Neurosciences and Cellular and Structural Biology Division, *Eunice Kennedy Shriver* National Institute of Child Health and Human Development, National Institutes of Health, Bethesda, MD 20892; 6 Quantitative Imaging and Tissue Sciences, *Eunice Kennedy Shriver* National Institute of Child Health and Human Development, National Institutes of Health, Bethesda, MD 20892

**Keywords:** myelin plasticity, synaptic plasticity, node of Ranvier, STDP, ocular dominance plasticity

## Abstract

Classic experiments showing that monocular visual disruption alters synaptic connections to binocular neurons in the brain established the fundamental concept of synaptic plasticity through coincident spike time arrival. However, if the speed of impulse transmission from the eye is altered by visual deprivation, spike time arrival at binocular neurons would be affected, thereby inducing synaptic plasticity. This possibility is tested here in adult mice by monocular eyelid suture and action potential inhibition in retinal axons. The results show that spike time arrival in visual cortex is altered by monocular visual disruption in association with morphological changes in myelin (nodes of Ranvier) on axons in optic nerve and optic tract. This non-synaptic mechanism of ocular dominance plasticity, mediated by myelin-forming cells, supplements and may drive synaptic plasticity.

## Introduction

A fundamental finding from the pioneering studies by Nobel Laureates Hubel and Wiesel (1) is that monocular visual deprivation (MD) alters synaptic strength and connectivity in neurons that receive input from both eyes, but binocular deprivation fails to have this effect (2). This form of synaptic plasticity is driven by differences in synchrony of spike time arrival from the two eyes onto neurons that receive binocular input.

This process applies more generally in neural networks where multiple inputs converge onto a common postsynaptic neuron. Synaptic inputs that arrive shortly after postsynaptic neuron action potential firing are weakened and inputs arriving just before or coincident with postsynaptic firing are strengthened. This basic synaptic learning rule (3) is termed spike-time dependent plasticity (STDP) (4,5).

What has been overlooked is that if the speed of impulse transmission is altered from one eye by visual deprivation, then by changing the synchrony of spike time arrival, axonal plasticity would influence synaptic plasticity and ocular dominance shifts (6). Moreover, changes in latency of spike time arrival will affect temporal summation of synaptic potentials in binocular neurons, potentially altering the waveform, latency, and amplitude of responses recorded in visual cortex. Since the relative amplitude of visually evoked potentials from the two eyes recorded in visual cortex is frequently used to monitor MD-induced ocular dominance shifts, the effects of spike time arrival on the waveform can be misinterpreted as exclusively reflecting changes in synaptic strength.

Neural impulse transmission speed is determined in part by the morphology of electrogenic nodes of Ranvier (NOR) along myelinated axons (7). Changes in NOR morphology (length of the nodal gap) and myelin thickness in optic nerve have been shown to alter neural impulse transmission speed, spike time arrival in the visual cortex, and visual acuity (8). This finding, in which nodal gap length was reversibly increased by inhibiting perinodal astrocyte exocytosis in transgenic mice, together with recent research on NOR plasticity in motor (9) and spatial (10) learning, and chronic stress (11) requires tests to determine whether visual disruption changes conduction velocity and NOR structure in optic nerve and optic tract.

The present studies address visual system plasticity in adult mice for several reasons. Less is known about visual system plasticity in adults than during early postnatal development, even though visual impairment in adults is a substantial medical problem. Moreover, plasticity mechanisms in adulthood can be applied more broadly to other neural circuits in mature animals. Many developmental processes in the early postnatal period are altered by use-dependent effects, including cell proliferation, differentiation, survival (12), cell migration and neurite outgrowth (13), synaptogenesis (14), synapse elimination (15), phagocytosis of synapses by microglia (16), and more, which operate through different cellular mechanisms. For review see (17). Similarly, visual deprivation in early life has multifaceted effects on the development of oligodendrocytes which form myelin in the optic nerve and optic tract (18) and in the developing cerebral cortex (19). These diverse developmental effects interact in complex ways to influence myelination, resulting in contradictory experimental outcomes and ambiguity. For review see (20).

Finally, structural remodeling of compact mature myelin is a comparatively slow process, requiring a week or more (8). In contrast, several mechanisms of synaptic plasticity proceed with much faster kinetics through changes in glutamate receptor expression (21, 22), inhibitory neuronal function (23, 24), and other physiological mechanisms that do not involve structural changes. Furthermore, the effects of prolonged visual deprivation have greater clinical relevance to ocular injury and disease.

The effect of MD on synaptic plasticity differs depending on whether visual deprivation occurs in the early postnatal period or in adults. MD in the early postnatal period rapidly changes synaptic strength in favor of the nondeprived eye by synaptic *depression* of inputs from the eye deprived of vision (1, 25). However, in adult mice subjected to MD imposed for longer than 5 days, visually evoked responses from the non-deprived eye are *strengthened* in the visual cortex ipsilateral to the open eye (25, 26). Two different types of synaptic plasticity are responsible for the different effects of MD in the early postnatal period and adults: synaptic competition and metaplasticity.

To determine whether visual deprivation alters NOR gap length in mature mice, monocular eyelid suture was performed on mice of both sexes at postnatal day 40 (P40) and NOR lengths on retinal ganglion cell (RGC) axons were measured in optic tracts by confocal microscopy at P70.

## Materials and Methods

### Materials

#### Mouse Strains

For Vesicular Glutamate Transporter 2 expressing (Vglut2+) retinal ganglion cells (27 ) with expression of AAV2-EF1α-FLEX-Kir2.1-T2A-tdTomato (28), mice were acquired from Charles River (C57BL/6 wild-type) and the Jackson Laboratory (Vglut2-ires-cre knock-in) (29, 30). All mice were housed in a 12-hr light/dark cycle with access to food and water ad libitum, following protocols approved by the NICHD/NIH Animal Care and Use Committee.

#### Plasmid Construct

Plasmid construct EF1α-FLEX-Kir2.1-T2A-tdTomato was gifted (31) and was previously created using pAAV-MCS and #60661 (Addgene) backbones (28). Plasmid DNA for packaging was obtained from Boston Children’s Hospital viral core facility.


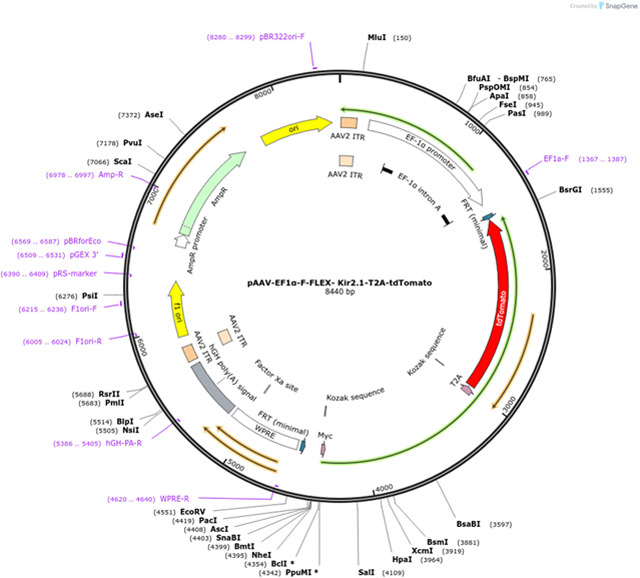


#### Virus Production

The resulting viral preparation, prepared by the NINDS Viral Production Core Facility, was an AAV serotype 2 vector encoding AAV2-EF1α-FLEX-Kir2.1-T2A-tdTomato, with a final titer of 7 μg.

#### Antibodies

Primary antibodies used were: Rabbit anti-Red fluorescent protein (1:200, Abcam, 124754); Guinea Pig anti-RBPMS (1:100, Phosphosolutions, 1832); Rabbit anti-enhanced green fluorescent protein (1:1000, Abcam, 290); Mouse anti-Cre recombinase (1:500, Millipore, 3120); Mouse anti-CASPR/Neurexin IV (K65/35) (1:50, UC Davis/NIH NeuroMab Facility, 73–001); Rabbit anti-Sodium Channel 1.6 (1:100, ASC009); Secondary antibodies used from Thermofisher Scientific, raised in mouse, rabbit or rat dependent on host species of primary antibody, highly cross absorbed and conjugated to fluorophores of Alexa Flour 488, 568, or 633 and used at 1:1000 dilution. For retinal nuclear staining, we used DAPI and Hoechst Nucleic Acid Stains (1:1000, Thermofisher, 62248).

### Methods

#### Monocular Deprivation

Animals were prepared the day before eyelid suture with carprofen for pain relief and continued for 2 days after the procedure. Animals were chemically restrained and placed under a microscope. The eyes are treated with Proparacaine Hydrochloride Ophthalmic Solution (USP 0.5%: NDC 61314–016-01) for discomfort relief and Ciprofloxacin Ophthalmic Solution (USP 0.3%.: NDC 61314–656-05) for antibacterial treatment. Triple Antibiotic Ophthalmic Ointment (TAOO) was used liberally on the sutures after surgery to prevent the eyes from drying out and to prevent infection. All ophthalmic solutions and chemical restraints used on animals were acquired through the NIH VDR. The animals’ eyelids were sutured together using 6–0 Nylon P-3 reverse cutting sutures (AD surgical) under strict sterile conditions. After surgery, the animals were monitored on a heating pad as they recovered post-operation, and until the animals recovered mobility to reach food and water. The animals were examined for tarsorrhaphy after 2 weeks.

#### Monocular Inhibition

Animals were chemically restrained with Ketamine (0.1 mg/g: NDC 11695-0703-1) and Xylazine (10 mg/kg NDC 59399-110-20) and placed under a dissecting microscope. The eyes were treated with Proparacaine Hydrochloride Ophthalmic Solution (USP 0.5%: NDC 61314–016-01) to manage pain. Cyclopentolate Hydrochloride Ophthalmic Solution (USP 1%: NDC 61314–396-03) and Phenylephrine Hydrochloride Ophthalmic Solution (USP 10%: NDC 42702–103-05) were administered to dilate the pupil to aid in visualization of the injection behind the lens. Once the animal was non-reactive to toe pinch, it was placed under a light microscope. The eye was proptosed, and a small opening was created on the side of the eye just above the retina using a 33-gauge beveled needle. The injection was done using a 38-gauge blunt needle inserted into the opening and guided behind the lens using the microscope to view the needle through the pupil. AAV (2μL at 1×10^1^ vg/mL of EF1α-FLEX-Kir2.1-T2A-tdTomato or CTB 488 or 555 to identify nodes of Ranvier by staining paranodes (2μL of 1 mg/mL, Fisher Scientific) at a rate of 200 nL/s. The needle position was held in place for 30 seconds before being slowly removed. The area was treated liberally with TAOO (NDC 16571–754-53) to prevent the eyes from drying out, and infection prevention, and the animals were placed in a clean cage on a heating pad to recover post-operation. All ophthalmic solutions and chemical restraints used on animals were acquired through the NIH Division of Veterinary Resources (VDR).

Patterned electroretinogram responses (pERG) were used to monitor inhibition of action potential firing from the retina as described in the electrophysiology section below.

The following experimental time course was used:


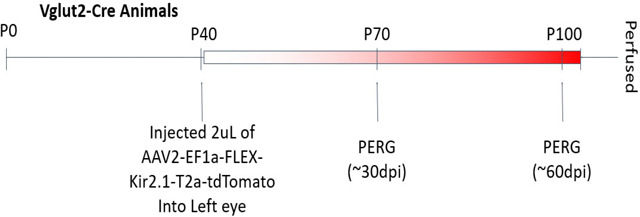


Transfection efficiency was calculated by determining the percentage of transfected retinal ganglion cells (RGCs) per field of view in the retinas. Total distributions of RGCs per field in Vglut2-Cre mice were comparatively analyzed by eye using an unpaired two-tailed Student’s *t*-test on mean values of confocal z-stack fields as described below.

#### Animal Perfusion and Tissue Preparation

For transcardial perfusion fixation, mice were deeply anesthetized under isoflurane using a VetEquip vaporizer until a toe pinch yielded no response. Anesthetized mice were secured to a perfusion platform on a downdraft table equipped with a chemical waste collection system. Following surgical exposure of the heart, a butterfly needle was inserted into the left ventricle for delivery of perfusion solutions, and a snip in the right atrium made to provide an outlet for perfusate. Gravity driven flow of phosphate buffer was perfused briefly (<5min) to clear blood from the vasculature, followed immediately by gravity-driven flow of 4% paraformaldehyde/PBS for light microscopy or 4% paraformaldehyde + 2.5% Glutaraldehyde/Cacodylate buffer for electron microscopy.

After fixation, optic nerves and tracts to be imaged by confocal microscopy were gently dissected from the brain and placed into 4% paraformaldehyde (PFA) (Electron Microscopy Sciences) in phosphate-buffered saline (PBS) overnight at 4°C and then transferred to 30% sucrose in PBS for 3 days. Optic nerve and optic tract issue was embedded in optimal cutting temperature (OCT) embedding medium (Fisher Scientific) and sectioned on a cryotome into 12 μm-thick slices and mounted on glass slides. Slides were stored at −20°C and rehydrated with PBS for immunocytochemistry.

#### Retinal Removal and Fixation

For immersion fixation for analysis of retinas, eyes were removed by proptosing eye with curved forceps immediately after euthanizing the animal, using small scissors to cleanly separate the eye from the optic nerve without stretching the optic nerve. Eyes were then transferred into 4% PFA for 15 minutes. Eyes were then transferred to PBS until dissection. Eyes were then transferred to a dish with a gel bottom, and fine dissection scissors were used to cut a circular hole around the cornea, taking care not to cut too deeply as to avoid cutting the retina. The cornea and lens were gently removed, leaving the retinal cup. Microdissection scissors were then used to make 4 to 5 symmetrical, radial cuts in the retinal cup, cutting through the retina and dark external epithelial and scleral layer. Cuts were made about 2/3 of the way down to the optic disc to allow the retinal cup to be flattened. Fine insect pins were used to hold all the pedals of the retinal cup flat, taking care to avoid puncturing the actual retina by pinning through the remaining iris at the periphery, or by gently lifting the retina away from its attachment to the sclera and pinning through the dark tissue underneath the retina. Pointed tweezers were used to gently split the retina and sclera, separating the connection of the peripheral edge of the retina to the underlying epithelial layer, repeating the process until all the edges of the retina were free. The free-floating retina was then gently transferred onto a glass slide, retinal cup-side up, and flattened using closed tweezers or a fine brush to unfold the pedals. Any visible debris on the retina was gently removed with tweezers, taking care not to damage the retina. A small square of filter paper was then placed over the flat retina to adhere it to the retina. The retina and filter paper were then transferred back into 4% PFA for a secondary 15-minute fixation. The retina on filter paper was then transferred into a fresh tube of PBS, and the retina was gently pushed off the filter paper so that it was free-floating in the PBS. The retina was then left to rinse for five minutes before starting the whole-mount immuno-staining protocol or stored in PBS at 4°C overnight if not starting the immunostaining process until the next day.

#### Immunostaining

For immunocytochemical staining of optic nerves and optic tracts, a blocking solution of 5% normal goat serum (Jackson ImmunoResearch), 0.5% Triton X-100 (Sigma-Aldrich), and 0.5% bovine serum albumin (BSA; Sigma-Aldrich) in PBS was applied for 2 h. Primary antibodies were diluted in the blocking solution and incubated overnight at 4°C. Slides were washed 3 times for 10 minutes with PBS and 0.5% Triton X-100 before secondary antibodies were applied for 2 h at room temperature at 1:1000 dilution in PBS, 0.5% Triton X-100, and 0.5% BSA. Secondary antibodies were centrifuged at 13k for 10 minutes at 4°C before application. Slides were washed 3 times for 10 minutes with PBS and 0.5% Triton X-100 and finally PBS alone for 10 minutes before being sealed with Prolong Diamond Antifade Mountant with DAPI (catalog #P36962, Thermo Fisher Scientific).

Retinas were lightly fixed in 4% paraformaldehyde (PFA) before and after retinal isolation and stored overnight in PBS at 4°C as described above. Retinas were rinsed with PBS for five minutes before adding blocking solution consisting of 5% normal goat serum (Jackson ImmunoResearch), 0.5% Triton X-100 (Sigma-Aldrich), and 0.5% bovine serum albumin (BSA; Sigma-Aldrich) in PBS, which was applied for at least one hour at room temperature with gentle shaking. Primary antibodies were diluted in the blocking solution and retinas were incubated for 48 hours at 4°C. Slides were washed 4 times for 10 minutes with PBS and 0.5% Triton X-100 before secondary antibodies were applied overnight at 4°C at 1:1000 dilution in PBS, 0.5% Triton X-100, and 0.5% BSA. Secondary antibodies were centrifuged at 13,000 rpm for 20 minutes at 4°C before application. A 1:1000 dilution of DAPI was added at the same time as secondary antibodies. Slides were washed 4 times for 15 minutes with PBS and 0.5% Triton X-100. Retinas were gently transferred from a tube to a slide, carefully avoiding pinching tissue. A drop of Prolong Diamond Antifade Mountant with DAPI (catalog #P36962, Thermo Fisher Scientific) was added to the retina on the slide.

#### Confocal Microscopy

Images were captured by confocal laser scanning microscope using Olympus Fluoview software (model Fluoview FV3000, Olympus) using an Olympus PlanApo N 60x/1.42 n.a. oil objective lens.

Nodes of Ranvier were immunohistochemical stained for Contactin Associated Protein 1 (Caspr1), a paranodal region-associated cell adhesion molecule expressed on the flanks of nodes of Ranvier, and by sodium channel Scn8a (Nav 1.6), which are concentrated within the nodal gap. Visualization of retinal tissue involved immunohistochemical staining for RNA-binding protein with multiple splicing (RBPMS) for retinal ganglion cells (RGC), and red fluorescent protein (RFP) to visualize viral transfection of RGCs, with DAPI staining to visualize cell nuclei.

#### Electron Microscopy

Mice were deeply anesthetized and perfused with 2.5% gluteraldehyde and 4% paraformaldehyde in 0.1 M sodium cacodylate, pH 7.4. Optic nerves were immediately removed from animals, then post-fixed in the same solution for 2 hr. Samples were post-fixed for 1 hr in 2% osmium tetroxide in 0.1M sodium cacodylate, pH 7.4. Samples were washed two times in 0.05 M sodium acetate, pH 5.0, and stained with 2% uranyl acetate in 0.05 M sodium acetate, pH 5.0, for 2 hr at 4°C. Samples were then dehydrated through a graded series of ethanol solutions followed by 100% acetone, and infiltrated with Epon 812 (Electron Microscopy Sciences, Hatfields, PA). Embedded samples were polymerized in an oven set at 60C. Ultrathin sections (90 nm) were cut with a diamond knife on a Leica EM UC7 Ultramicrotome. Sections were placed on 200 mesh copper grids and post-stained with, uranyl acetate and lead citrate and examined by transmission electron microscopy on a JEOL-1400 Transmission Electron Microscope operating at 80 kV and with an AMT BioSprint-29 megapixel camera with 9.5 micron square pixels.

All images were coded and analyzed without the knowledge of the investigator making the measurements. The diameter of all axons cut in cross section was measured, both for the axon itself and the fiber diameter including the myelin sheath. Transected axons are rarely perfect circles, so both the major and minor axes of each cross sectioned axon were measured and averaged to calculate the g-ratio as the axon diameter divided by the fiber diameter. Measurements of axon diameter, fiber diameter, number of axons, and g-ratios of all axons in the field of view were averaged and used for statistical testing, comparing between experimental and control conditions using unpaired two-tailed Student’s *t*-tests. This design avoids pseudo-replication that would result from using each axon as the sample size in statistical test calculations.

#### Image Acquisition and Nodal Gap Measurement

In both the monocular deprivation and monocular inhibition paradigms, an Olympus PlanApo N 60x oil microscope objective (60x/1.42 n.a. Oil; UIS2 BFP1; ∞/0.17/FN26.5) with Olympus IMMOIL-F30CC immersion oil type-f was used at a 4x zoon. Olympus Fluoview software was used for image acquisition and analysis of optic nerve and tract samples. A z-series of ten sections of 0.2 μm intervals was taken for optic nerve samples for the monocular inhibition paradigm, and a z-series of 30 sections at 0.2 μm intervals was taken for optic tract analysis, for both the monocular deprivation and monocular inhibition experiments with the opposite eye of the same animal used as a control. Thicker samples were required in the optic tract because axons are less highly compacted than in the optic nerve. Ten regions of interest along the full length of the optic nerve or tract were imaged at random locations. A single-plane maximum projection of the z-series stack was created using Olympus Fluoview and NIH ImageJ software. In samples with virus-infected RFP+ axons, a single-plane maximum projection was not created, and each stack was reviewed one image at a time to ensure co-localization of RFP+ axons and Caspr1. Nodal gap lengths were measured using NIH ImageJ software by using the line measurement tool to measure from the edge of one Caspr1-labeled paranode to the opposing paranode edge delimiting the nodal gap. The mean nodal gap for all nodes of Ranvier in the field of view was calculated and used for statistical analysis, rather than using each node of Ranvier as n = 1. A total of 35,560 nodes of Ranvier from a total of 6,600 individual images were used for this study.

In the monocular inhibition paradigm, retinal ganglion cell (RGC) numbers were counted in the retinas of postnatal day 100 (P100) animals to ensure that the virus was not significantly altering the number of RGCs and to determine the proportion of cells overexpressing Kir2.1. Flat-mount retinas were stained with anti-RNA-binding protein with multiple splicing (RBPMS) antibodies and DAPI to visualize total RGC numbers and to confirm that a signal in the image is a cell. If a cell had both RBPMS signal and was RFP+, a RGC has been successfully infected with the virus. Flat-mount retina images were captured by a confocal laser scanning microscope with an Olympus UPlanSApo 20x microscope objective (20x/0.75; UIS2; ∞/0.17/FN26.5) at a 1x zoom using the Olympus Fluoview software. A z-stack of 30 sections at 0.2 μm intervals was collected at five random points in the retinas. A single-plane maximum projection was formed for each flat-mount retina image and the point tool was used to count individual cells and record whether they are RFP+. A total of 29,885 RGCs were counted from the retinas of N=3 Vglut2-Cre animals of both sexes.

### Electrophysiology

#### Pattern Electroretinogram (pERG) and Pattern Visual Evoked Potential (pVEP)

Animals were chemically restrained using Ketamine (0.1 mg/g) and Xylazine (10 mg/kg). The animals were placed on the testing platform for recording (Diagnosys Celeris apparatus) which maintains body temperature of 37°C. Animals were treated prior to pVEP and pERG with Proparacaine Hydrochloride Ophthalmic Solution (USP 0.5%:: NDC 61314–016-01) for discomfort of the probe on the eye. Cyclopentolate Hydrochloride Ophthalmic Solution (USP 1%:: NDC 61314–396-03) and Phenylephrine Hydrochloride Ophthalmic Solution (USP 10%:: NDC 42702–103-05) were administered to dilate the pupil. All ophthalmic solutions and chemical restraint used on animals were acquired through the NIH VDR.

Visual stimulation using bars of 50cd/m^2^ intensity was delivered by a miniature LCD display placed directly on the corneas. Horizontal bar patterning was presented to the animals, with 4 bars in the image and a rotation of 2 cycles per degree. Two sets of 300 pERG and pVEP runs were recorded and averaged. Each eye was tested individually. The animals were positioned on the platform with the stimulator on each eye. The ground lead was placed into the hind flank of the animals, and the recording lead for pVEP was placed subdermally on the skull above the visual cortex at the midline. The pattern stimulator was placed on the eye being tested, and an unused full-field stimulator was placed on the other eye as an electrical control. Stimulation information was recorded using diagnosys Espion V6 software. The animals’ eyes were treated with TAOO to prevent drying. After recording, the mice were placed on a warming pad in a clean cage for recovery. Animals were monitored until mobility was restored.

Latencies and amplitudes of pVEP in wild-type monocularly deprived mice were compared between both eyes using unpaired two-sample Student’s *t*-tests. The ratio of latency to peak pVEP from the two eyes from the expected ratio of 1 were analyzed using one-sample Student’s *t*-tests. Peak amplitudes of pERG from Vglut2-Cre mice were compared between transfected and untransfected eyes using unpaired two-sample Student’s *t*-tests.

### Statistical Analysis

All samples were coded and analyzed by a different investigator without knowledge of the experimental condition. Statistical analysis of all data was done using GraphPad Prism (v10.0). Normality was assessed using the Shapiro-Wilk or Kolmogorov-Smirnov test for the data reported. All data were reported as mean ± SEM, and two-sided statistical comparison was utilized (unless otherwise noted) and considered significant at p < 0.05. To assess whether differences in pVEP latency between the two eyes of individual animals differed significantly from the expected ratio of 1, a one-tailed t-test was used. This approach eliminated variation in electrophysiological data among different animals by using each animal as its control.

Pseudo-replication was avoided in nodal gap length and ultrastructural data by averaging the values within confocal z-stack or EM fields, with each field being treated as an independent replicate. Unpaired two-tailed Student’s *t*-tests were used to compare the experimental conditions for nodal gap length paradigms. To compare the density of nodal structures by lateral localization in the optic nerves and optic tracts of monocularly inhibited Vglut2-Cre animals, an unpaired two-tailed Student’s *t*-test was used to test for differences between axons from the two eyes of the same animal. In this design, each animal serves as its own control, avoiding the added variance from cross-animal comparisons. Nodal densities were calculated by taking the total number of nodes per field and comparing the respective optic nerves and optic tracts.

## Results

### Morphometric Measurement of Nodes of Ranvier

**Fig. 1.**
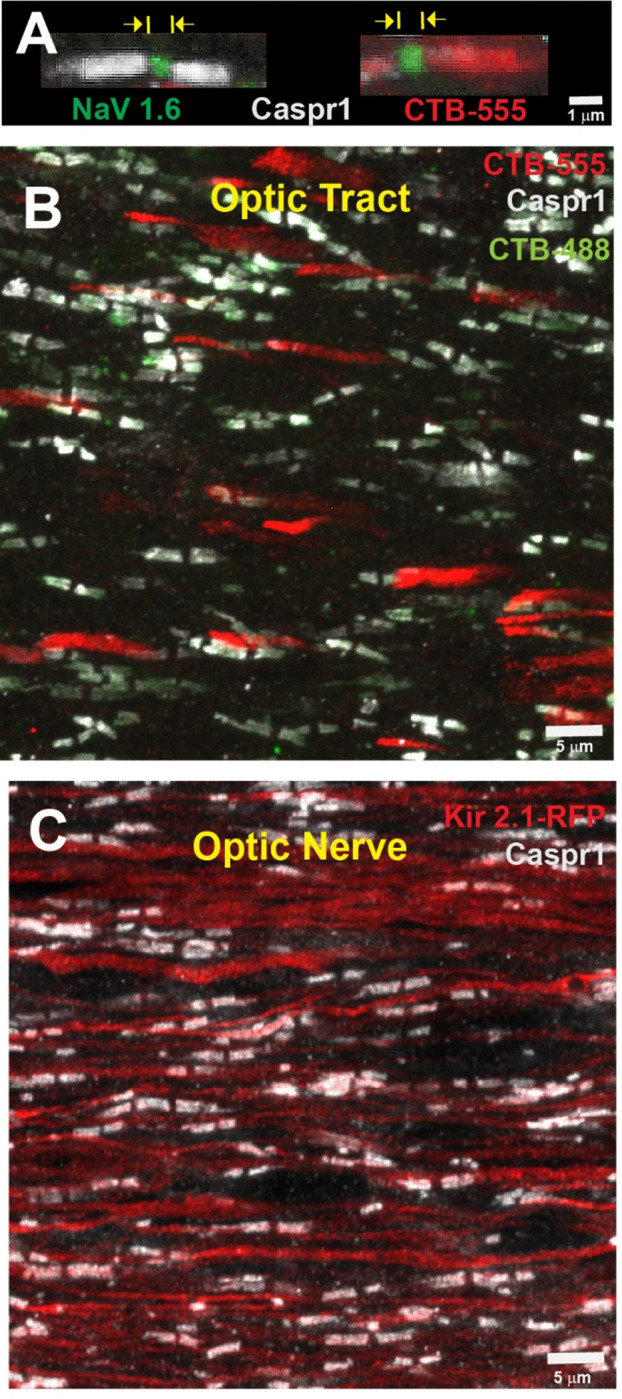
Nodes of Ranvier in mouse optic nerves and tracts after monocular deprivation and monocular inhibition. **(A)** Nodes of Ranvier (NOR) were identified by using antibodies for Contactin-associated protein 1 (Caspr1) to label paranodes (grey), and sodium channels SCN8A (NaV1.6) within the nodal gap (green). Intravitreal injection of AlexaFluor 555-conjugated cholera toxin subunit B (CTB-555), which accumulates in the paranodal regions, tags nodes on axons originating from the sutured eye (red). Nodal gap lengths were measured from paranode to paranode for all nodes in the field of view. (**B**) Intraocular injection of CTB-555 into the sutured eye (red), and CTB-488 into the open eye (green) was used to identify whether NOR were on optic tract axons from the deprived eye or open eye. (**C**) To inhibit action potential firing in a subset of axons in optic nerve, AAV2-EF1a-FLEX-Kir2.1-T2A-tdTomato was transfected into one eye and those axons were identified by the red fluorescence (Kir 2.1 RFP).

Nodes of Ranvier were identified using confocal microscopy and immunohistochemical identification of Contactin-associated protein 1 (Caspr1) in the paranodal region, and sodium channels Nav1.6 in the nodal gap (Fig. 1A). Intraocular injection of the green fluorescent dye cholera toxin B conjugated to AlexaFluor-488 (CTB-488) into the open eye and red fluorescent cholera toxin B conjugated to AlexaFluor-555 (CTB-555) into the eye with eyelid suture was used to identify whether nodes were on axons from the sutured or unsutured eye (Fig. 1B). Thirty Z-plane stacks of 53 X 53 μm^2^ confocal fields of view were collected at 0.2 μm intervals and projected onto a single plane for analysis. This represents a sampled depth of 6.6 μm, and a volume of 0.185 mm^3^ considering the approximately 0.8 μm depth of a single confocal image plane. Nodal gap lengths were measured from paranode to paranode flanking the nodal region for all nodes in the single-plane projection. The mean nodal gap length of all NOR in this volume was taken as n = 1 for statistical testing, with 10 such microscope fields quantified in randomly chosen locations along the length of each optic tract in multiple experimental replicates. This conservative procedure avoids pseudoreplication that would artificially increase the sample size if every node of Ranvier measured in these experiments (a total of 35,560 nodes of Ranvier) was used as the sample size in statistical test calculations (32). A total of 6,600 individual images were analyzed. This conservative experimental design increases confidence in the positive findings.

### Plasticity of Nodes of Ranvier in Optic Tracts Following MD

**Fig. 2.**
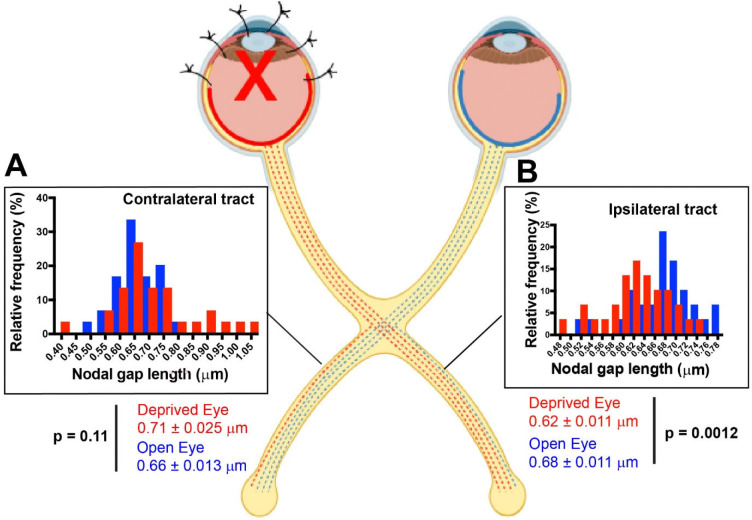
Monocular deprivation by eyelid suture of adult mice alters the length of nodes of Ranvier in optic tracts. Nodal gaps were significantly shorter on axons from the deprived eye (red) compared to axons from the uninhibited eye (blue) in the optic tract ipsilateral to the open eye (p = 0.0012, t-test_(df = 58)_ = 3.403). In the contralateral optic tract, there was a strong trend for nodes to be longer on axons from the deprived eye (red) relative to axons from the open eye (blue) in the same tract (p = 0.11, t-test_(df = 58)_ = 1.618), but the differences failed to reach statistical significance at the p < 0.05 probability level. Nodal size frequency histograms are shown for nodal gap lengths measured on axons averaged by field from the sutured eye (red) and unsutured eye (blue) for the contralateral (**A**) and ipsilateral optic tracts (**B**).

The results showed that NOR were significantly shorter on optic tract axons from the deprived eye compared to axons from the unsutured eye in the optic tract ipsilateral to the unsutured eye (p < 0.003, t-test_(58 df)_ = 3.12) (Fig. 2). An opposite effect was apparent in the contralateral tract, with a strong trend for NOR on axons from the deprived eye to be longer than those from the open eye. However, the statistical probability of the difference was below the p = 0.05 threshold criterion (p = 0.11, t-test_(58 df)_ = 1.62) (Fig. 2).

The different effects of NOR plasticity between the ipsilateral and contralateral optic tracts parallels the different types of synaptic plasticity predominating in ipsilateral and contralateral visual cortex of adult mice (26). MD by eyelid suture for 12 days in mice between the ages of P60–90 leads to persistent NMDA receptor-dependent enhancement of visual evoked potential (VEP) responses from the open eye in the visual cortex ipsilateral to the open eye, but little or no change in deprived eye responses in that cortex (25, 26).

The synaptic strengthening of afferents from the non-deprived eye in ipsilateral cortex results from metaplasticity, in which the threshold and direction of synaptic strength modification depends on the prior level of ongoing postsynaptic activity (33,34). Retinal axons from the eye deprived of vision fire spontaneously, but they lack the coordinated firing of axons driven by visual experience. The relatively stochastic synaptic input from the deprived eye is not coincident with postsynaptic action potential firing and the level of postsynaptic activity is reduced. Since 90% of retinal axons decussate to the opposite side of the brain in mice (35), the ongoing level of patterned neural activity is greatly reduced in visual cortex ipsilateral to the open eye after MD, thus lowering the threshold for synaptic potentiation and strengthening synapses from the open eye.

Metaplasticity does not predominate in visual cortex contralateral to the open eye, because afferent activity is reduced in only 10 percent of the axons. Here the effects are variable, depending on the interplay between STDP and metaplasticity, which vary with experimental parameters; notably postnatal age and the duration of visual deprivation (25, 26). Synaptic competition predominates in young mice, thereby increasing responses to the open eye relative to the deprived eye in both cortices; however this process abates in older mice after the close of the critical period. Whether changes in myelin also occurred in optic tract axons in association with metaplasticity during monocular deprivation was unknown.

The finding of significantly shorter NOR on axons from the MD eye in ipsilateral optic tract reveals a form of NOR plasticity that is compatible with metaplasticity during ocular dominance changes in adults. This raises the question of whether neural transmission speed is altered in axons from the visually deprived eye.

### Effect of MD on Spike Time Arrival in Visual Cortex

Mathematical modeling and electrophysiological measurements in mouse optic nerve show that lengthening the nodal gap by 0.14 μm results in approximately 20% slower action potential transmission speed, and 6.7 ms increased latency of spike time arrival at the visual cortex (8). This difference in spike time arrival falls within the temporal window of spike time arrival synchrony with respect to postsynaptic action potential firing to induce STDP plasticity (36–38). A nodal gap length increase of this magnitude is functionally significant, with a reduction in visual acuity of about 0.01 cycles per degree (8). Also, in wild-type rat optic nerve and cortex, NOR gap lengths vary over a 4.4 and 8.7-fold range respectively in each tissue, and mathematical modeling predicts that these nodal length differences will alter conduction speed by ~20% (39). The significantly shorter NOR gap length on axons from the deprived eye in the optic tract ipsilateral to the open eye would predict an increase in conduction velocity in these axons.

To determine whether MD alters spike time arrival in the visual cortex, the latency to peak visually evoked potential using patterned visual stimulation (pVEP) was determined by inserting a subcutaneous recording electrode against the skull above the visual cortex at the midline. After 30 days MD, sutures were removed from the deprived eye under dim red light to preserve dark adaptation in mice, which lack L-opsin photoreceptors and thus cannot detect red wavelengths (40). Using a miniature video display placed on the corneas, computer-generated visual stimulation was then provided sequentially to each eye while maintaining animals in the dark-adapted state. The LED video screen displayed 4 horizontal bars with 50 cd/m^2^ intensity illumination, sweeping the visual field vertically at 2 cycles per degree. Electrophysiological cortical responses from 600 sweeps were averaged.

**Fig. 3.**
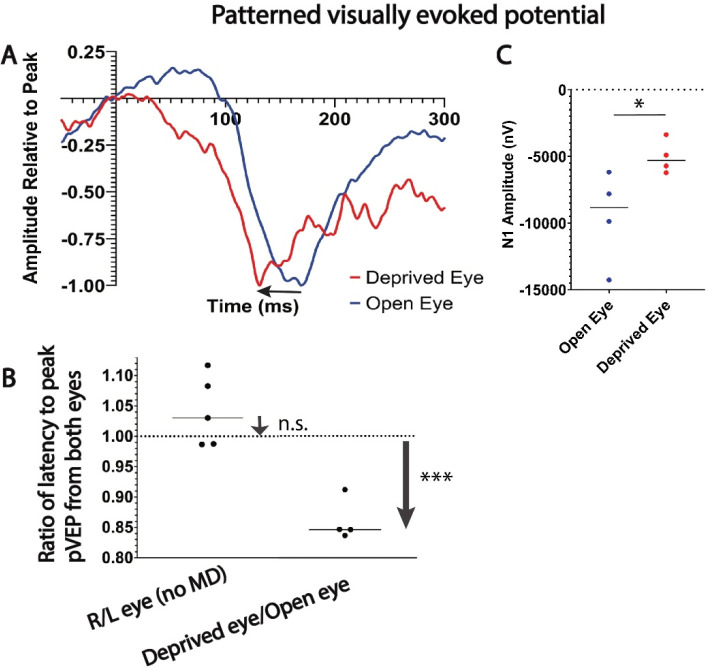
Monocular deprivation reduces the latency and amplitude of visually evoked potentials in visual cortex from the deprived eye. (**A**) Representative patterned visually evoked potential waveforms from the unsutured eye (blue trace) and previously sutured eye (red trace) of an adult mouse, showing the shortened latency to peak pVEP (arrow) from the eye that had been deprived of vision for 30 days. (**B**) The ratio of latency to peak pVEP from the two eyes was not significantly different from an expected ratio of 1 in mice with normal visual experience (ratio of 1.04 right eye/left eye ± 0.06, p = 0.19, one-tailed t_(4 df)_ = 1.575), but was significantly reduced from the deprived eye (ratio of 0.86 ± 0.03 MD/open eye, t_(3 df)_ = 8.040; p = 0.004), consistent with faster conduction velocity in MD axons. (**C**) The amplitude of pVEP responses were smaller from the eye that had been sutured (p = 0.05, two-sample t_(6 df)_ = 2.410). *p < 0.05; *** p < 0.0004, n.s. non-significant.

The measurements showed that the latency to peak pVEP was shorter in every animal from the deprived eye compared to their unsutured eye, with a mean decrease in latency of 13.9% from the deprived eye of mice that had undergone 30 days MD (p = 0.004, t-test_(3 df)_ = 8.040) (Fig. 3). Comparing latencies of VEP responses from both eyes of the same animal eliminates inter-animal variability in VEP response latencies. As expected, in mice that did not experience MD, the ratio of latencies to peak pVEP response from the two eyes using each animal as its own control was not significantly different from a ratio of 1 (p = 0.19, one-tailed t-test _(4 df)_ = 1.575) (Fig. 3B). In addition, the mean amplitude of pVEP evoked by the deprived eye (−5057 ± 1750 nV) was about half that of the nondeprived eye (−9535 ± 624.8 nV), (p = 0.05, t-test_(6 df)_ = 2.410) (Fig. 3C). This is consistent with depression of synaptic strength from axons originating from the retina deprived of vision. Thus plasticity of NOR gap length after MD in adult mice changes conduction velocity in a manner that parallels plasticity of synaptic strength under similar conditions.

### Oligodendrocytes as Mediators of Action Potential Synchrony

Plasticity of NOR on RGC axons and spike time arrival in visual cortex following MD expands understanding of ocular dominance plasticity beyond the well-established synaptic plasticity mechanisms, but there is a fundamental difference. In contrast to synaptic plasticity where coincident activity is detected by postsynaptic neurons, in NOR plasticity, spike synchrony in the optic tract must be detected by oligodendrocytes, which are situated along the axon, far from synaptic terminals. Similarly, as it is currently understood, metaplasticity depends on the general level of postsynaptic activity, but this information is not directly accessible to the myelin forming cells.

Recently a theory of oligodendrocyte mediated plasticity (OMP) has been proposed to address this problem (41). A single oligodendrocyte forms myelin through up to 50 slender cell processes extending from the soma. Each process wraps a segment of myelin independently around a different axon. Action potentials trigger localized intracellular calcium responses in oligodendrocyte processes at sites of axon contact through the vesicular release of glutamate from axons activating mGluR and NMDA receptors on oligodendrocytes. This intercellular signaling stimulates activity-dependent myelination by increasing local translation of myelin basic protein (42). Thus, action potentials in axons are detected at contacts between the oligodendrocyte’s individual processes associated with different axons. If the timing of spikes arriving among the population of axons that are myelinated by a single oligodendrocyte initiate changes in myelin on each axon to minimize temporal delays in spike time arrival among the local population of axons that the oligodendrocyte contacts, then mathematical modeling indicates that the synchrony of spike time arrival at the axon terminal is greatly improved (41). In this way, the oligodendrocyte could act in an analogous manner to the postsynaptic neuron in assessing synchrony of action potentials, but rather than evaluating the relative timing of postsynaptic potentials from synapses converging onto the postsynaptic neuron, the oligodendrocyte evaluates the synchrony of action potentials among the population of axons that it myelinates in transmitting information to the terminal. Likewise, the total action potential output of axons myelinated by a single oligodendrocyte could influence nodal plasticity, similar to how cumulative postsynaptic activity governs metaplasticity.

The visual system anatomy provides a way to test the hypothesis that activity-dependent differences in spike time arrival among individual axons myelinated by an oligodendrocyte could drive adaptive changes in NOR morphology. While each optic nerve carries input from only one eye, the optic tracts (located beyond the optic chiasm where nerves partially decussate) contain axons from both eyes. Thus, oligodendrocytes have access to neuronal traffic from both eyes in the optic tracts, but not in the optic nerves. Recent research shows that 30 days of binocular visual deprivation in adult mice does not alter nodal gap length in optic nerve significantly (43) and does not affect recovery of nodal gap length after it is lengthened through inhibition of perinodal astrocyte exocytosis (44). These findings parallel synaptic plasticity in the visual system, which is driven by monocular, but not by binocular deprivation.

**Fig. 4.**
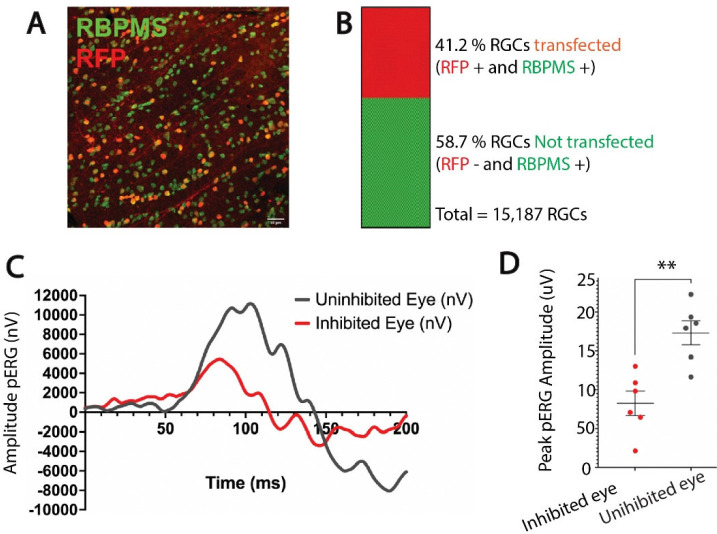
Overexpression of the inwardly rectifying potassium channel 2.1 reduces amplitude of pattern electroretinogram responses (pERG). (**A**) Representative confocal microscope field of retinal ganglion cells in the monocularly inhibited retina of adult mice measured at postnatal day 100. Retinal ganglion cells were labeled with antibodies for RNA-binding protein with multiple splicing (RBPMS) (green). Red fluorescent protein (RFP) (red) indicates retinal ganglion cells transfected with Kir2.1. (**B**) Retinas from the eyes injected with AAV2-EF1a-FLEX-Kir2.1-T2A-tdTomato had an average of 41% of the RGC transfected with Kir2.1 to inhibit action potential firing (red), and 58% were not transfected (green) N = 3 mice. (**C**) Representative electroretinogram response to patterned visual stimulation showing greatly reduced responses after inhibiting action potentials in approximately half of the RGCs of one eye (red), compared to responses in the eye other eye in the same mouse that was not transfected (black). (**D**) Mean peak pERG amplitudes were significantly reduced in retinas after Kir2.1 transfection compared to uninhibited retinas (4123 ± 786.9 nV vs. 8672 ± 777.6 nV; p = 0.0021 t-test_(10 df)_ = 4.112). * p < 0.05; ** p < 0.005. There was no significant difference in the total number of retinal ganglion cells between the monocularly inhibited eye and the uninhibited eye (p = 0.6, t-test_(28 df)_ = 0.4868).

To test the hypothesis that NOR plasticity is driven by differences in activity among axons in the visual pathway, a mixture of functionally active and inactive axons was imposed in the optic nerve to mimic the situation in optic tract. To achieve this, neural impulses were suppressed in a subset of axons in optic nerve by transfecting inwardly rectifying potassium channel (Kir2.1) selectively into retinal ganglion axons of one eye by intraocular injection of AAV2-EF1a-FLEX-Kir2.1-T2A-tdTomato (45). Histological analysis of retinas showed potassium channel Kir2.1 expression in approximately 41.3% of RGCs (Fig. 4 A, B). Electrophysiological recording of pattern electroretinograms (pERGs) confirmed that retinal responses were significantly attenuated through the course of the experiment by the overexpression Kir2.1 to inhibit action potential firing in RGCs (p = 0.0021; t-test_(10 df)_ = 4.112) (Fig. 4 C, D).

**Fig. 5.**
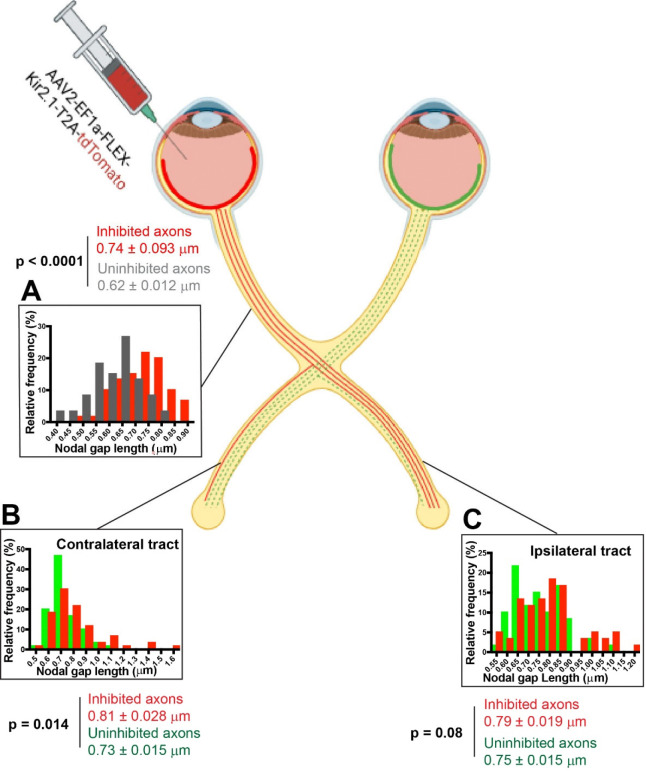
Inhibiting action potential firing in a subset of axons from one eye, reveals an activity-dependent axon-specific mechanism of nodal plasticity. **(A)** In the optic nerve, nodes of Ranvier were significantly larger on axons transfected with Kir2.1 to inhibit action potentials (red) compared to untransfected axons (grey) in the same nerve (p = 0.0001, t-test_(118 df)_ = 6.41). (**B**) In the optic tract contralateral to the untransfected eye, nodes of Ranvier were significantly larger on axons with action potentials inhibited (red) compared to untransfected axons (green) in the same tract (p = 0.014, t_(118 df)_ = 2.48). (**C**) A similar trend was observed in the optic tract ipsilateral to the open eye, with larger nodes of Ranvier on axons with action potentials inhibited (red) compared to untransfected axons (green) in the same nerve, but the differences did not reach the 0.05 level for statistical significance (p = 0.08, t-test(_118 df)_ = 1.76).

The results using Kir2.1 transfection into RGCs showed that when oligodendrocytes in optic nerve are provided access to a mixed population of axons with either visually-evoked activity or inhibited action potential activity, activity-dependent differences in NOR length resulted which were axon-specific (Fig. 5). Nodal gap length was increased selectively on optic nerve axons in which action potentials were inhibited by expressing Kir2.1 (p = 0.0001, t-test _(118 df)_ = 6.41).

Monocular inhibition (MI) of action potential firing also resulted in lengthening NOR gaps on axons with inhibited action potential firing in the optic tract contralateral to the open eye (p = 0.014, t-test _(118 df)_ = 2.48) (Fig 5). A similar effect was apparent in the ipsilateral optic tract (p = 0.08, t-test _(118 df)_ = 1.76), but the differences failed to reach p < 0.05 level of statistical significance.

Together these results reveal that in addition to modification of synaptic strength in the lateral geniculate nucleus and visual cortex, MI and MD induce axon-specific activity-dependent changes in NOR gap length that will affect the speed of impulse transmission to the CNS. This is consistent with an axon-specific mechanism of NOR plasticity that is dependent on differential levels of neural impulse activity in individual axons, rather than a tissue-level effect, as for example could result from activity-dependent release of neurotrophic factors.

### Ultrastructural Differences Following MI

In addition to changes in NOR gap length, several other factors contribute to spike time arrival in visual cortex, including synaptic delays, myelin thickness, axon diameter, and ion channel composition in the nodes. Previous studies have shown no differences in axon diameter or myelin thickness in optic nerve following visual deprivation (18). Possible ultrastructural differences in optic nerve axons after MI were analyzed in the present experiments by transmission electron microscopy using the same experimental animals used for NOR analysis by confocal microscopy.

**Figure 6.**
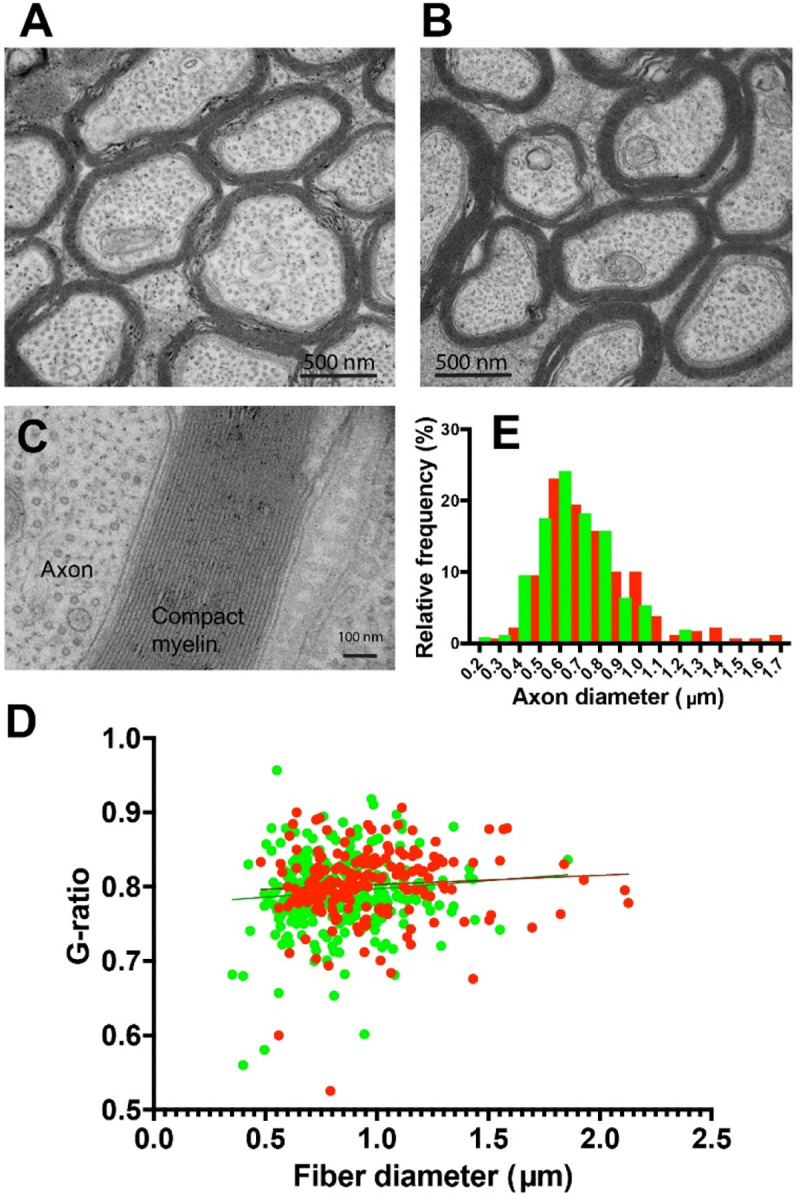
Ultrastructure of axons in optic nerve after MI by viral transfection of Kir2.1 to inhibit action potential firing. Normal appearing ultrastructure of axons in the optic nerve from the transfected eyes (**A**), compared to the untransfected eyes (**B**). (**C**) High magnification showing normal compaction of myelin after MI. (**D**) No significant difference in myelin thickness (g-ratio) in transfected optic nerve axons after MI (red) compared to axons in the untransfected eyes (green), mean 0.80 ± 0.0041 vs 0.79 ± 0.0038 transfected (red) vs untransfected (green) eyes, (p = 0.20, t-test_(57 df)_ = 1.29). Plot shows 192 axons from the transfected optic nerve and 288 axons from the untransfected nerve. (**E**) The mean caliber of axons was slightly larger in the MI condition 0.79 ± 0.19 μm vs 0.68 ± 0.18 μm, transfected (red) vs untransfected eye (green), (p = 0.0001, t-test_(55 df)_ = 4.136).

At the ultrastructural level, axon morphology and myelin appeared normal in optic nerves after MI, with no evidence of axon degeneration (Fig. 6 A-C). Quantitative analysis revealed no significant difference in mean myelin thickness as expressed by g-ratio in the two optic nerves of the same mice after MI inhibition (g-ratio = 0.80 ± 0.0042 vs 0.79 ± 00038, inhibited nerve vs uninhibited nerve (p = 0.20, t-test _(57 df)_ = 1.295) (Fig. 6 D).

There were, however, fewer axons in the optic nerve transfected with Kir 2.1 (16.5 ±0.75 vs 19.5 ± 0.6 axons/29.7 μm^2^, p < 0.003, t-test _(57 df)_ = 3.07), and consequently, fewer NOR in the optic nerves transfected with Kir 2.1 (80.4 ± 2.59 vs. 95.9 ± 3.18 nodes/7303μm^3^, p < 0.0002, t-test_(118 DF)_ = 3.80). Since the control eye was injected with CTB-488 tracer, the difference in number of axons is not likely caused by the injection, but rather a result of Kir2.1 expression. This suggests that in addition to activity-dependent competition and metaplasticity affecting NOR gap length on axons from the retinas, there is also a deprivation-dependent process of axonal atrophy after prolonged inhibition of action potentials in optic nerves of adult mice. There is little prior research on possible loss of axons after prolonged action potential inhibition in adults, but during development, activity-dependent access to trophic factors and activation of apoptotic pathways eliminates ineffectual axons and neurons (46 for review).

A small but significant increase in mean axon caliber was also evident by electron microscopy in optic nerve axons from the eye experiencing MI (0.79 ± 0.019 vs 0.68 ±0.18 μm, inhibited nerve vs. uninhibited nerve, p = 0.0001, t-test _(55 df)_ = 4.13) (Fig. 6E). There is no relation, however, between nodal gap length and axon caliber in optic nerve (39). In theory, increased axon caliber would tend to increase conduction velocity in transfected axons, but the question is largely moot, as action potential firing is inhibited in these axons. A limitation of the electron microscopy data is that unlike NOR measurements by confocal microscopy, where individual axons from each eye are labeled with different fluorescent indicators, the ultrastructural data in optic nerve from the MI eye represent mean values comprised of a mixture of axons with action potentials inhibited by Kir2.1 transfection, and axons with normal visually evoked activity.

## Discussion

The implicit assumption in studies of ocular dominance plasticity that the speed of impulse transmission is the same from both eyes and is not altered by visual experience is not supported by these experimental results. The data show that MD alters the latency of spike time arrival in visual cortex, with shorter latency visually-evoked potentials from the eye deprived of vision by eyelid suture for 30 days in adulthood. This is accompanied by morphological plasticity of nodes of Ranvier, resulting in shorter nodal gaps on axons from the deprived eye in optic tract ipsilateral to the open eye.

An axon-specific mechanism of NOR plasticity is demonstrated by the significantly different NOR gap lengths on axons from the eye deprived of vision by eyelid suture compared to axons from the non-deprived eye in optic tracts. The lack of changes in NOR length in optic nerves of mice deprived of binocular vision for 30 days (43, 44) suggests a plasticity mechanism dependent upon differences in activity among individual axons. Confirming this experimentally, when differences in activity among axons in optic nerve were introduced by inhibiting action potentials in a subset of axons by transfection of Kir2.1, nodal gap length was increased specifically on the inhibited axons. This supports a competitive activity-dependent mechanism of NOR plasticity as predicted by OMP theory (41).

Thus, upstream of synaptic plasticity detected in the CNS in ocular dominance studies, there is an axon-specific process of activity-dependent plasticity in the optic nerve and tract. NOR plasticity will affect action potential arrival time at binocular neurons, the coincidence of excitatory post-synaptic potentials (EPSPs) with respect to postsynaptic firing, temporal summation of postsynaptic potentials, and thereby the overall level of depolarization and action potential firing in the postsynaptic neurons. Enlarged nodes on axons slows conduction velocity and by STDP this will promote depression of synapses from these axons, whereas shorter nodal gaps speed impulse transmission and this would promote synapse potentiation. In addition, there was a small but significant decrease in the number of axons in optic nerves transfected with Kir2.1, which is consistent with an atrophic effect from suppressing neural activity for 30 days in adult mice occurring in tandem with the other mechanisms of plasticity.

Overall the findings suggest that oligodendrocytes serve to modify the transmission of information in axons in an activity-dependent manner analogous to the postsynaptic neuron in mediating activity-dependent synaptic competition (47) and metaplasticity (33, 48). Both synaptic and nodal plasticity emphasize the importance of synchrony of information transmission in activity-dependent plasticity, with synchrony of postsynaptic potentials assessed by the postsynaptic neuron and synchrony of action potentials in transmission to the target assessed by oligodendrocytes.

The effects of MD on NOR morphology have not been investigated previously. Prior research has shown that during early postnatal development in mice, monocular eyelid suture has several other effects, including increasing differentiation of oligodendrocytes and shortening the mean length of myelinated segments between NOR (18). Curiously, the opposite effect on oligodendrogenesis is observed following optogenetic stimulation of mouse motor cortex, however (49). The possible developmental effects of MD on NOR gap length were not investigated in the study by Etxeberria et al., (18). In contrast to the axon-specific effects on NOR gap length found in the present studies on adult mice, the changes caused by MD during postnatal development and myelin formation were not specific to the axons from the deprived eye, but also extended via an unknown mechanism to surrounding axons conveying normal vision.

Comparing the present results on NOR plasticity during ocular dominance plasticity with similar studies of synaptic plasticity raises several questions. The effects of visual deprivation on ocular dominance plasticity depend on the experimental parameters used, notably the species and age of the animal and the duration and means of visual disruption. Experimental paradigms used to study ocular dominance plasticity vary widely, making cross comparisons with the present study more difficult. Transfection of Kir2.1 was used in the present studies to provide an effective and long-lasting inhibition of action potential activity in RGC axons. Studies of inhibiting action potential activity on ocular dominance plasticity frequently use intraocular tetrodotoxin (TTX) injection to block voltage-dependent sodium channels, but this requires repeated injections to sustain inhibition (25). This approach is not feasible for the present experimental objectives. Our preliminary experiments measuring pERG responses of retinal excitation showed that intraocular TTX injections inhibited retinal activity in mice for less than 24 hrs. Repeated injections to achieve 30 days of MI are not feasible, due to the trauma caused by daily injections. For this reason, prior studies of prolonged MI were limited to 3 daily TTX injections in adult mice for an experimental time-course of 5 days (25). Moreover, Kir2.1 transfection achieves the experimental objective of suppressing the level of action potential firing in a subset of axons rather than blocking action potentials from retinas as in experiments using TTX.

The new findings also raise methodological issues that complicate interpretation of previous research. Ocular dominance studies frequently take the ratio of VEP amplitudes that are evoked by patterned visual stimulation of the two eyes to monitor ocular dominance shifts (50). This is a reliable indicator, but difficult to interpret at a cellular level. The ratio of VEP amplitudes can change by either increases or decreases in the numerator, denominator, or both. Mechanistically, changes in VEP ratios can result from depression and/or potentiation of synapses from either eye.

However, the evoked potentials are population responses reflecting the combined synaptic currents, and differences in latency of spike time arrival will affect the peak, amplitude, and latency of the VEP, complicating interpretation of any differences in VEP ratio. Prior research has interpreted changes in the ratio of VEP amplitude evoked by the deprived and non-deprived eye as ocular dominance shifts due to synaptic plasticity, and this mechanism is firmly established by mechanistic studies; notably by the involvement of the NMDA receptor (21, 22). The changes in NOR morphology and conduction velocity in the visual pathway identified here could affect VEP responses by promoting synaptic potentiation and depression, through STDP and metaplasticity. In addition, the primary data can be confounded by changes in the VEP waveform that can result from differences in spike time arrival due to NOR plasticity and other factors affecting impulse conduction velocity in the optic nerve and tract. Changes in VEP waveform and latency to peak are not typically measured in ocular dominance studies. The present findings suggest the possibility that changes in spike time arrival, as a consequence of NOR plasticity and other potential axonal changes, should be considered in future research.

Activity-dependent changes in NOR in other contexts suggest that just as synaptic plasticity operates through different mechanisms in association with development, pathology, physiological regulation, and experience-dependent modification, NOR plasticity also occurs in distinct contexts, operating through different cellular mechanisms. Large changes in nodal gap length can be produced during NOR remodeling in development, in demyelinating disorders (51), after non-physiological intense stimulation, or stressful conditions. For example, nodal gap length is greatly increased in auditory nerve following hyperstimulation after prolonged intense sound (52), and large changes in NOR gap length are also increased after prolonged sleep deprivation (53). These changes increase the probability of conduction failure and would seem to reflect pathological responses.

The size of the experience-dependent changes in NOR gap length in the present studies, while tenths of a micron, is substantial relative to the approximately one-micron size of nodes of Ranvier. Changes in NOR gap length of this magnitude would adjust conduction velocity within the normal range to influence spike time arrival and physiological function (8). Nodal gap plasticity of this scale is produced biologically by reversibly severing the attachment of myelin sheaths to the axon via the cell adhesion molecule neurofascin-155 in the paranodal region (54). NOR gap changes in this size range have previously been reported following spatial learning (10) and motor learning (9). Nodes of Ranvier much longer than one micron can lead to severe impairment of impulse conduction as seen in demyelinating diseases (55).

## Conclusion

These new findings reveal a previously unknown contributor to experience-dependent plasticity operating through changes in action potential conduction velocity and myelin plasticity. Other mechanisms that affect conduction velocity could cooperate in shortening the latency of spike time arrival in visual cortex from the deprived eye; however, changes in NOR morphology and spike time arrival in visual cortex after MD open a new avenue for investigation in ocular dominance plasticity. These findings advance our understanding of ocular dominance plasticity and, more broadly, in other neural circuits where synaptic modification is driven by the spike time arrival of axonal inputs converging onto a common postsynaptic target. Additionally, these findings carry translational relevance for therapeutic strategies involving monocular visual disruption and disease.

## Data Availability

Available upon request.
